# Optimal Frequency for Cranial Electromagnetic Field Stimulation

**DOI:** 10.7759/cureus.81436

**Published:** 2025-03-29

**Authors:** Alice S Wang, James Brazdzionis, Paras Savla, Raphia K Rahman, Dan E Miulli

**Affiliations:** 1 Neurosurgery, Riverside University Health System Medical Center, Moreno Valley, USA; 2 Neurosurgery, Arrowhead Regional Medical Center, Colton, USA

**Keywords:** atraumatic brain injury, electromagnetic field frequency, electromagnetic field stimulation, neuronal circuit, tramatic brain injury

## Abstract

Background

Communication among neurons generates electromagnetic fields (EMFs) that can be measured through a noninvasive, portable helmet equipped with 20 sensors. The EMF data reveal a variety of EMF patterns that have yet to be elucidated. Understanding a propagated frequency from the brain and its subunits can assist with diagnosing the brain and its subunits’ function and treatment. Here, the authors provide an interpretation of the EMF patterns with an emphasis on frequency.

Methods

From January 2025 to February 2025, a prospective clinical study was conducted to enroll patients greater than 18 years old diagnosed with atraumatic and traumatic brain injury whose EMFs, which were collected using a helmet equipped with 20 sensors, were obtained within 24 hours of presentation. EMF data were collected using DAQami software (DATAQ Instruments Inc., Akron, Ohio, United States) and analyzed using fast Fourier transformation with Igor Pro 8 software (WaveMetrics Inc., Lake Oswego, Oregon, United States). Based on each patient’s clinical presentations and/or radiographic findings, the sensors of interest, their opposing sensors, and frequencies of interest (FOIs) were selected.

Results

A total of 10 patients were enrolled with a mean age of 47.1 years. Mechanisms of injury included spontaneous hypertensive intracranial hemorrhage (one patient) and head trauma after a motor vehicle collision, dirt bike accident, or ground-level fall (nine patients). Radiographic findings included spontaneous basal ganglia hemorrhage (one patient), isolated traumatic subdural hematoma (one patient), traumatic subarachnoid hemorrhage (one patient), and no intracranial abnormalities (seven patients). The following targeted FOIs were found: 5.2 Hz, 7.3 Hz, 7.6 Hz, 7.7 Hz, 7.9 Hz, 8.3 Hz, 8.6 Hz, 8.7 Hz, 9.5 Hz, and 10.4 Hz.

Conclusions

EMF of the human brain reveals changes in neuronal activities in atraumatic and traumatic brain injury patients. This information allows for the localization of sites of brain injuries and the selection of frequencies that can be used for understanding the EMF frequency and function on the macroscopic level as well as at the cellular level. This specific information can then be utilized for stimulation to modulate the changes in neuronal, circuit, and brain function activities. Our frequency selection technique enables more precise, tailored, and potentially more effective treatment aiming to restore EMF activity.

## Introduction

One neuron communicates with another one in an organized fashion, serving as the basis of any neural circuitry. The electric and chemical transmissions among the neurons generate electromagnetic fields (EMF), which represent the summation of all the electrochemical activities. Many factors, such as head trauma, can cause injury to any part of a neuron (dendrite, cell body, axon, and synaptic cleft), leading to ischemia, vasogenic edema, mitochondrial dysfunction, reactive oxygen species, neurotoxicity, apoptosis, inflammation, and/or diffuse axonal injury [1,2]. Subsequently, the changes may affect a neuron’s ability to send electrical signals or release neurotransmitters to effectively communicate with another neuron, impacting different areas of the brain and the overall synchronization of the brain. The neural circuitry may become misaligned, manifesting as changes in waveforms on EMF when compared to another neural circuitry in which the neurons are functioning properly.

The waveforms depicted as frequencies and amplitudes of EMF are correlated to different levels of consciousness. Delta waves, ranging from 0.0 Hz to 4.0 Hz, are typically observed in states of diminished consciousness. Theta waves, ranging from 4.0 Hz to 6.0 Hz, play a role in motor function and speech processing. Alpha waves, ranging from 8.0 Hz to 13.0 Hz, are the hallmark electrophysiologic rhythm of the normal and awake brain [3-7]. Similarly, based on prior studies, the authors observed fewer peaks and valleys and decreased oscillations in comatose patients compared to more peaks and valleys and increased oscillations in awake patients in the frequency range from 0.0 Hz to 4.0 Hz. They also observed fewer peaks and valleys from 6.0 Hz to 12.0 Hz in patients with clinical symptoms without correlating radiographic findings such as in the setting of concussion compared to more peaks and valleys and increased oscillations in normal patients in the same frequency range. Based on these observations, the EMF recordings of the human brain are grouped into one of three categories: Category 1, which represents 0.0-4.0 Hz for those in a comatose state; Category 2, which represents 4.0-6.0 Hz for those with neurologic deficits of neuronal circuits (i.e., speech, motor, and/or sensory deficits); and Category 3, which represents 6.0-12.0 Hz for those suffering from concussion-like symptoms or cognitive deficits (e.g., headache, loss of consciousness, and difficulty with concentration) without basic functional neurologic deficits of Category 2 on examination [8,9].

A noninvasive portable helmet has been used to perform EMF recordings and stimulations [8-13]. The authors previously demonstrated successful localization of abnormal brain EMF activity to correlate with neurological symptoms and deficits and/or structural lesions on the CT head, providing an insight into changes in the neuronal cells, neural circuitry, and neuronal pathways [9]. Modulation of EMF activity via stimulation has been investigated in animal models with traumatic brain injury, spinal cord injury, and/or global transient stroke [12-16]. In a swine model with controlled cortical impact, it was established that EMF stimulation using thresholds of 2.5 Hz and 5.5 Hz resulted in improvement of EMF patterns on treatment day 7 [13]. These specific frequencies likely correlate to certain neuronal changes such as gene and protein expression or myelination [17]. In humans, a recent study investigated the combination of transcranial direct current stimulation (tDCS) and modified constraint-induced movement therapy for improving motor recovery in post-stroke patients and found that tDCS intervention at 2 mA or 4 mA did not facilitate motor recovery when compared to sham, suggesting that higher doses of tDCS intervention may be necessary [18].

The authors hypothesize that the EMF patterns that may be of relevance include a pair of sensors displaying opposite polarity, a peak, and a valley at a specific frequency. At this frequency, the sensor spanning over the site of brain injury displays a valley, which represents a lower amplitude, when compared to the sensor spanning over the site of no brain injury, which displays a peak, which represents a higher amplitude. When both sensors show similar directionality of their amplitudes, this suggests the electrochemical signaling flow in a similar direction at the same frequency. However, when there is a change in the directionality of both sensors, one becoming a valley and the other becoming a peak, this demonstrates the electrochemical signaling is flowing in opposite directions at the same frequency. This change may be due to disruptions in receptor signaling, ion flow, myelination, or neurotransmitter release at this frequency. Clinically, these disruptions at this frequency may manifest as symptoms of concussion such as headache, dizziness, and memory issues; symptoms of abnormal function such as weakness, lack of sensation, and language issues; or symptoms of consciousness. Therefore, by targeting EMF stimulation at a specific frequency, the goal is to realign the electrochemical flow to stabilize, improve, and possibly restore the normal function of the neuronal cell, circuit, and overall brain function to allow for conscious and subconscious thoughts to be processed properly. Successful restoration of diseased states through EMF stimulation may then manifest as a resolution of headache, dizziness, memory issues, sleep changes, behavior changes, function, and consciousness.

Although EMF stimulation has been investigated in animal models of TBI, the optimal parameters for EMF modulation, particularly the ideal stimulation frequency, remain unknown in the human population [12-14]. In this article, the authors aim to describe how to select the ideal frequency for EMF stimulation to induce maximal brain modulation in patients with brain injury.

## Materials and methods

Study design

Our institution’s institutional review board approved this prospective study (Arrowhead Regional Medical Center, Protocol #23-58). Inclusion criteria included patients greater than 18 years old with head trauma or intracranial hematomas. Exclusion criteria included a Glasgow Coma Scale (GCS) of 3, contraindications for donning a helmet such as active hemodynamic or respiratory instability, or refusal of study enrollment. Two portable racks equipped with horizontal rods and four cords were used to suspend the helmet in the air, and the cords provided adjustable tension to securely hang the helmet in place just above the patient’s head. A portable, lightweight helmet with shielding constrained to a dual-layered Mu-metal (MuMETAL, Magnetic Shield Corporation, Bensenville, Illinois, United States) and copper layering and engineered with Mu-metal 18-inch channels to place sensors and EMF signal generators (BS-1000, Quasar Federal Systems, San Diego, California, United States) was built to allow for EMF recording at bedside instead of bringing the patient to a room designed for EMF recordings. The sensors and EMF signal generators were placed in a specific configuration. In this paper, each sensor’s spanning region is clarified in parenthesis; for example, sensor 7 (left frontal lobe). The sensors were placed in the channels 9 inches away from the scalp, providing a 6.37-degree field of view. The known spatial relationship between the sensors allowed for identifying regions of overlap or opposite configurations, where sensors in opposing positions (180 degrees from each other) were expected to demonstrate opposite polarities for a specific EMF. Each sensor was also positioned with the positive end oriented toward the scalp.

EMF data collection and analysis

**Figure 1:**
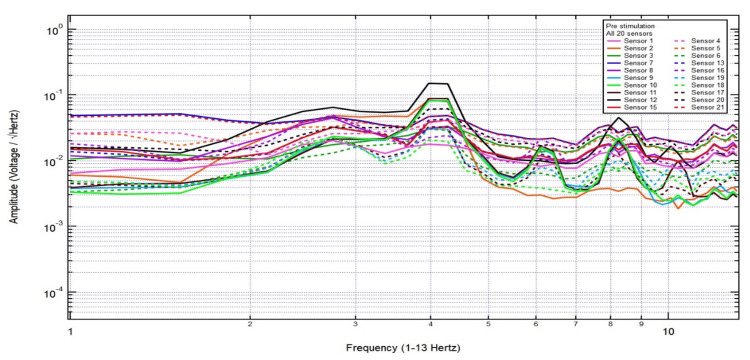
Example of EMF recordings from all 20 sensors EMF, electromagnetic field

EMF recordings at 5,000/second in each sensor were collected using DAQami software (DATAQ Instruments Inc., Akron, Ohio, United States) and analyzed using fast Fourier transformation (FFT) with Igor Pro 8 software (WaveMetrics Inc., Lake Oswego, Oregon, United States). Each EMF recording was 30 seconds. To ensure stability of data, only 20 seconds of the recording was used for analysis (data between five seconds after the start of recording and five seconds before the end of the recording), yielding 100,000 points of data from each sensor. The sensors recorded up to 2,500 Hz. In this paper, the authors investigated up to 13.0 Hz. Thus, graphs of EMF recordings from 1.0 to 13.0 Hz were then generated using FFT with Igor Pro 8 software, with the x-axis representing frequency and the y-axis representing the summation of the voltage amplitude as the square root of the frequency. Figure 1 illustrates an example of the EMF recordings of all 20 sensors.

Based on each patient’s clinical presentations and radiographic findings, one pair of sensors out of the ten pairs was selected. In the selected pair of sensors, the sensor spanning the site of brain injury was termed the targeted sensor of interest (SOI), and the sensor located on the opposite side was termed the opposing sensor (OS). From the targeted SOI and OS, the frequencies displaying opposite polarity within each patient’s assigned category were identified, and they were termed frequencies of interest (FOIs). Opposite polarity was defined as a valley found in the SOI and a peak found in the OS. A valley was at a lower amplitude than a peak, suggesting some deficits in brain activity. If there was only one FOI, then this FOI was selected and termed as the targeted FOI. If there were multiple FOIs, then the lowest frequency was chosen as the first targeted FOI.

## Results

This study included 10 patients with a mean age of 47.1 years (range: 23-81 years). Mechanisms of injury included spontaneous hypertensive intracranial hemorrhage (one patient) and head trauma after a motor vehicle collision, dirt bike accident, or ground-level fall (nine patients). Radiographic findings included spontaneous basal ganglia hemorrhage (one patient), isolated traumatic subdural hematoma (one patient), traumatic subarachnoid hemorrhage (one patient), and no intracranial abnormalities (seven patients). The following targeted FOIs were found: 5.2 Hz, 7.3 Hz, 7.6 Hz, 7.7 Hz, 7.9 Hz, 8.3 Hz, 8.6 Hz, 8.7 Hz, 9.5 Hz, and 10.4 Hz (Table 1).

**Table 1:** Patient demographics, clinical presentations, CT findings, and EMF findings EMF, electromagnetic field; FOI, frequency of interest; LOC, loss of consciousness; GCS, Glasgow Coma Scale; SOI, sensor of interest; OS, opposing sensor

Patient	Age in years/sex	Mechanism of injury	Chief complaint	Clinical findings	CT findings	Category (frequency range in Hz)	EMF findings: targeted SOI (OS)	FOI
1	38/male	Motor vehicle collision	LOC	GCS15 left frontal scalp hematoma	No intracranial abnormalities	3 (6.0-12.0 Hz)	20 (12)	8.3 Hz
2	23/male	Motorcycle accident	Right posterior temporal headache, helmeted, LOC	GCS15, headache 2/10	No intracranial abnormalities	3 (6.0-12.0 Hz)	11 (17)	8.6 Hz and 11.6 Hz
3	23/female	Motor vehicle collision	Right frontotemporal headache, no LOC	GCS15, headache 5/10	No intracranial abnormalities	3 (6.0-12.0 Hz)	8 (16)	7.7 Hz
4	36/male	Dirt bike accident	Right shoulder pain, head strike, no LOC	GCS15	No intracranial abnormalities	3 (6.0-12.0 Hz)	12 (20)	7.3 Hz
5	81/female	Ground level fall	Bilateral frontal headache, head strike, LOC	GCS15, bilateral forehead ecchymosis	Right subarachnoid hemorrhage in the ambient cistern	3 (6.0-12.0 Hz)	21 (15)	7.6 Hz
6	46/male	Motor vehicle collision	Left top headache, head strike, LOC	GCS15, headache 8/10	No intracranial abnormalities	3 (6.0-12.0 Hz)	6 (3)	7.9 Hz
7	81/male	Ground level fall	Left temporal headache, head strike, LOC	GCS15, headache 10/10, right frontal scalp hematoma	No intracranial abnormalities	3 (6.0-12.0 Hz)	17 (11)	8.7 Hz
8	57/male	Motor vehicle collision	Right posterior temporal headache, head strike, no LOC	GCS15, headache 6/10	Anterior falx subdural hematoma	3 (6.0-12.0 Hz)	11 (17)	7.9 Hz, 9.5 Hz, and 11.4 Hz
9	62/male	Hypertension	Hard to speak, right arm weakness, no LOC	GCS15, expressive aphasia, 4/5 right upper extremity motor strength, right pronator drift	Acute left basal ganglia hemorrhage	2 (4.0-6.0 Hz)	5 (2)	5.2 Hz
10	24/male	Motorcycle accident	Left frontal headache, head strike, LOC	GCS15, headache 7/10	No intracranial abnormalities	3 (6.0-12.0 Hz)	21 (15)	10.4 Hz

Patient 1

**Figure 2:**
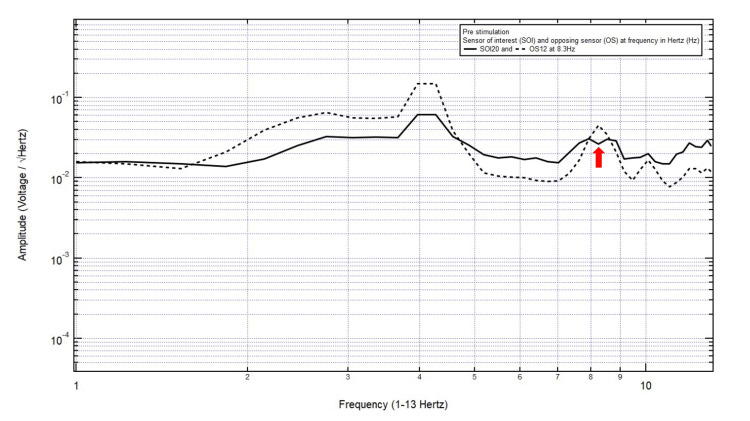
EMF of Patient 1 showing a single pair of peaks and valleys at 8.3 Hz (red arrow) EMF, electromagnetic field

A 38-year-old male presented with a chief complaint of headache and dizziness after a motor vehicle collision with loss of consciousness. The patient was GCS15 with a small left frontal scalp hematoma, thus localizing the site of brain injury to SOI20 (left frontotemporal region) with its OS12 (right parietooccipital region). Within Category 3, only one pair of peaks and valleys was observed at 8.3 Hz, shown with the red arrow in Figure 2. Therefore, 8.3 Hz was the selected targeted FOI (Table 1).

Patient 2 

**Figure 3:**
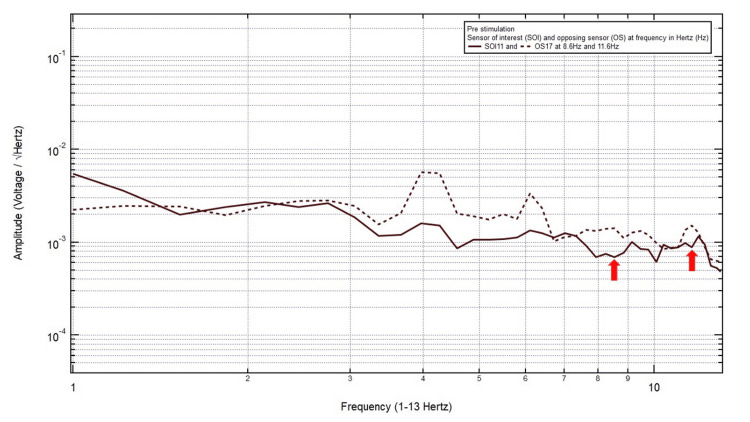
EMF of Patient 2 showing two pairs of peaks and valleys at 8.6 Hz and 11.6 Hz (red arrows) EMF, electromagnetic field

A 23-year-old male presented with a chief complaint of a right posterior temporal headache after a motorcycle accident, helmeted, with loss of consciousness. The patient was GCS15 and had a right posterior temporal headache, localizing the site of brain injury to SOI11 (right posterior temporal region) with its OS17 (left posterior temporal region). Within Category 3, two pairs of peaks and valleys were observed at 8.6 Hz and 11.6 Hz, shown with the red arrows in Figure 3. In numerical order, 8.6 Hz was selected as the first targeted FOI. If the patient did not show improvement clinically and electromagnetically at this targeted FOI after EMF stimulation, then 11.6 Hz would be the next targeted FOI.

Patient 3 

**Figure 4:**
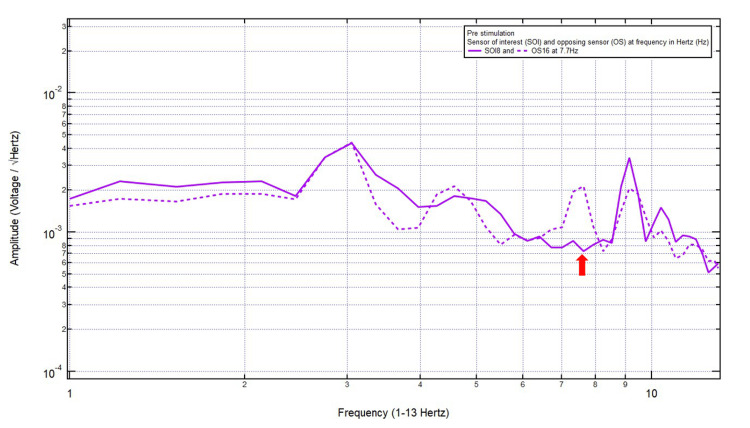
EMF of Patient 3 showing a single pair of peaks and valleys at 7.7 Hz (red arrow) EMF, electromagnetic field

A 23-year-old left-handed female presented with a chief complaint of a right frontotemporal headache after hitting the right side of her head in a motor vehicle collision without loss of consciousness. The patient was GCS15 and had a right frontotemporal headache localizing the site of brain injury to SOI8 (right frontotemporal region) with its OS16 (left parietooccipital region). Within Category 3, only one pair of peaks and valleys was observed at 7.7 Hz, shown with the red arrow in Figure 4. Therefore, 7.7 Hz was selected as the targeted FOI.

Patient 4

**Figure 5:**
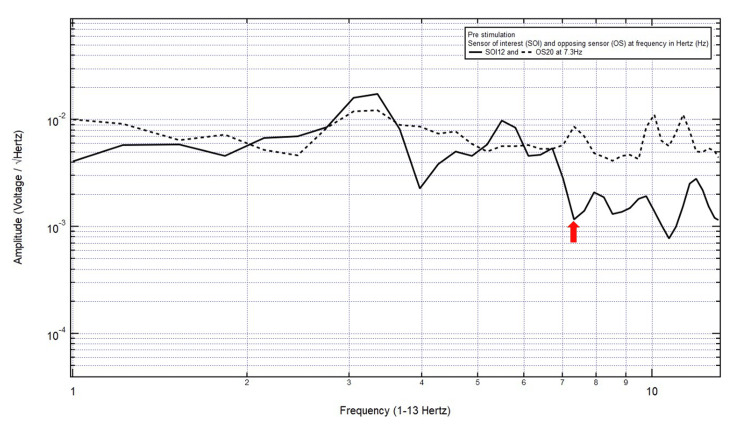
EMF of Patient 4 showing a single pair of peaks and valleys at 7.3 Hz (red arrow) EMF, electromagnetic field

A 36-year-old male presented with a chief complaint of right shoulder pain after a dirt bike accident, helmeted, with a head strike, and without loss of consciousness. The patient was GCS15 with right shoulder pain, localizing the site of brain injury to SOI12 (right parietooccipital region) with its OS20 (left frontotemporal region). Within Category 3, only one pair of peaks and valleys was observed at 7.3 Hz, shown with the red arrow in Figure 5. Therefore, 7.3 Hz was selected as the targeted FOI.

Patient 5

**Figure 6:**
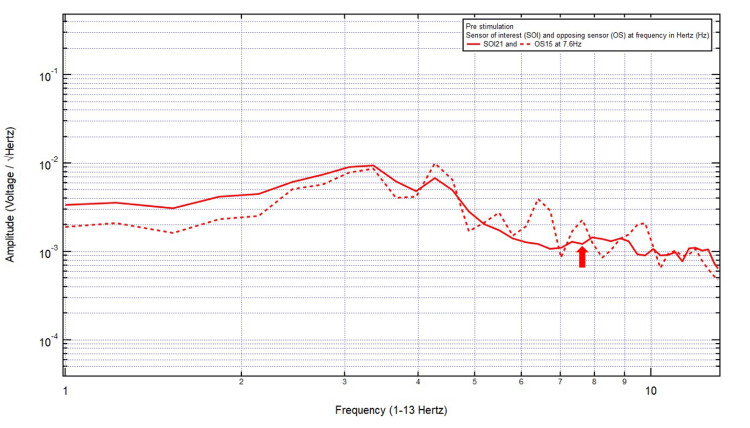
EMF of Patient 5 showing a single pair of peaks and valleys at 7.6 Hz (red arrow) EMF, electromagnetic field

An 81-year-old female presented with a chief complaint of bilateral frontal headache, 1/10 in pain, after a ground-level fall with a head strike and loss of consciousness. The patient was GCS15 with bilateral forehead ecchymosis, localizing the site of brain injury to SOI21 (left frontal lobe) with its OS15 (left occipital lobe). Within Category 3, only one pair of peaks and valleys was observed at 7.6 Hz, shown with the red arrow in Figure 6. Therefore, 7.6 Hz was selected as the targeted FOI.

Patient 6

**Figure 7:**
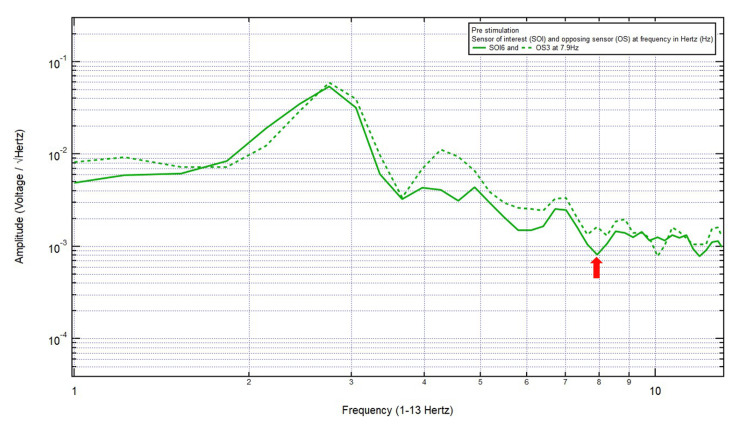
EMF of Patient 6 showing a single pair of peaks and valleys at 7.9 Hz (red arrow) EMF, electromagnetic field

A 46-year-old left-handed male presented with a chief complaint of a left top headache, 8/10 pain, after a motor vehicle collision with a head strike and loss of consciousness. The patient was GCS15 and had left eye ecchymosis and facial swelling, localizing the site of brain injury to SOI6 (left motor cortex and deeper structures) and OS3 (right sensory cortex and deeper structures). Within Category 3, only one pair of peaks and valleys was observed at 7.9 Hz, shown with the red arrow in Figure 7. Therefore, 7.9 Hz was the selected targeted FOI.

Patient 7 

**Figure 8:**
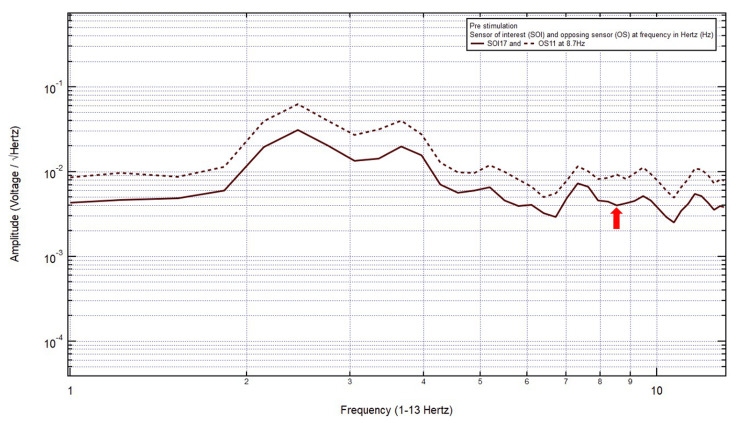
EMF of Patient 7 showing a single pair of peaks and valleys at 8.7 Hz (red arrow) EMF, electromagnetic field

An 81-year-old male presented with a chief complaint of a left temporal headache, 10/10 pain, after a ground-level fall with a head strike and loss of consciousness. The patient was GCS15 and had a large right frontal scalp hematoma, localizing the site of brain injury to SOI17 (left posterior temporal region) with its OS11 (right posterior temporal region). Within Category 3, only one pair of peaks and valleys was observed at 8.7 Hz, shown with the red arrow in Figure 8. Therefore, 8.7 Hz was selected as the targeted FOI.

Patient 8 

**Figure 9:**
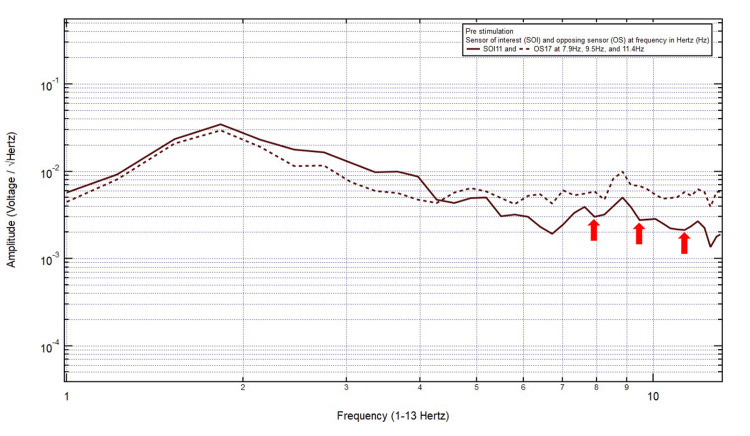
EMF of Patient 8 showing three pairs of peaks and valleys at 7.9 Hz, 9.5 Hz, and 11.4 Hz (red arrows) EMF, electromagnetic field

A 57-year-old right-handed male presented with a chief complaint of right posterior temporal headache after falling back with a head strike after being hit by a truck from the left side without loss of consciousness. The patient was GCS15 and had a right posterior temporal headache with 6/10 pain. CT head showed an anterior falx subdural hematoma without midline shift. EMF data showed localization to SOI11 (right posterior temporal region) and OS17 (left posterior temporal region). Within Category 3, three pairs of peaks and valleys were observed at 7.9 Hz, 9.5 Hz, and 11.4 Hz, shown with the red arrows in Figure 9. In numerical order, 7.9 Hz was selected as the targeted FOI. If the patient did not show improvement clinically and electromagnetically at this targeted FOI after EMF stimulation, then 9.5 Hz would be the next targeted FOI, followed by 11.4 Hz as the final targeted FOI.

Patient 9 

**Figure 10:**
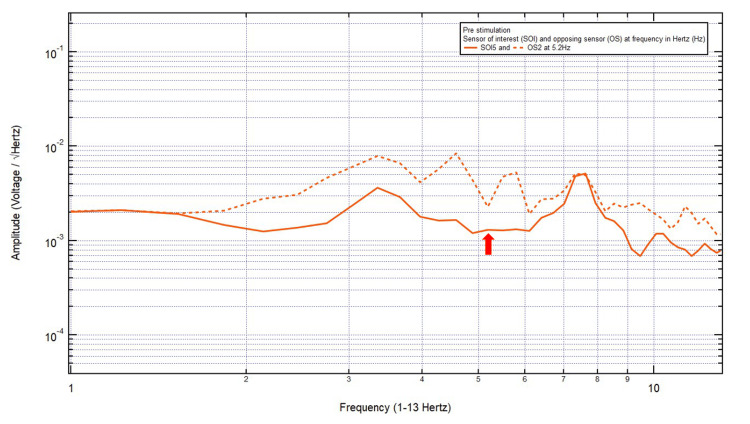
EMF of Patient 9 showing a single pair of peaks and valleys at 5.2 Hz (red arrow) EMF, electromagnetic field

A 62-year-old male presented with a chief complaint of acute aphasia and right upper arm weakness. The patient was GCS15 and had expressive aphasia, 4/5 on the right upper extremity, and right pronator drift. CT head showed an acute left basal ganglia hemorrhage. EMF data showed localization to SOI5 (left sensory cortex and deeper structures) with its OS2 (right motor cortex and deeper structures). Within Category 2, only one pair of peaks and valleys was observed at 5.2 Hz, shown with the red arrow in Figure 10. Therefore, 5.2 Hz was the selected targeted FOI.

Patient 10 

**Figure 11:**
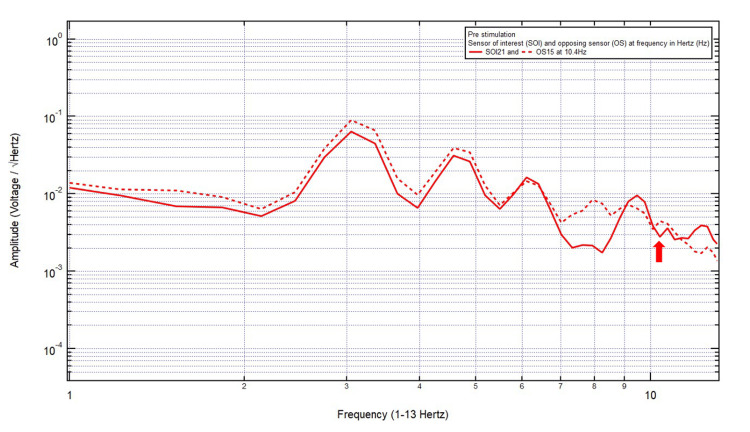
EMF of Patient 10 showing a single pair of peaks and valleys at 10.4 Hz (red arrow) EMF, electromagnetic field

A 24-year-old male presented with a chief complaint of a left frontal headache, 7/10 pain, after a motorcycle accident, helmeted and with loss of consciousness. The patient was GCS15 with localizing the site of brain injury to SOI8 (right frontotemporal region) and OS16 (left parietooccipital region). Within Category 3, only one pair of peaks and valleys was observed at 10.4 Hz, shown with the red arrow in Figure 11. Therefore, 10.4 Hz was the selected targeted FOI.

## Discussion

Using a noninvasive, portable helmet equipped with 20 sensors, the authors were able to measure EMF up to 2,500 Hz in patients. The EMF data demonstrated that the same chief complaint was not associated with the exact frequency. However, the frequency changes did correspond to the appropriate categories of changes: Category 1 (0.0-4.0 Hz) for those in a comatose state, Category 2 (4.0-6.0 Hz) for those with neurologic deficits of neuronal circuits, and Category 3 (6.0-12.0 Hz) for those suffering from concussion-like symptoms or cognitive deficits [8,9]. For example, many of the patients with concussion complained of headaches, and their EMF data did not all show opposite polarity at the same frequency. Most of the opposite polarities were found between 7.0 Hz and 9.0 Hz within Category 3, which ranged from 6.0 Hz to 12.0 Hz. The implication of this finding suggests that not all concussion-induced headaches have the same effect on the electrochemical flow in the neural circuitry. This makes sense given the underlying causes of headaches are multifactorial and may be due to variations in disruption of the anatomical structures such as the meninges, severity of the mechanism of injury in various areas, differences in age, and medical conditions such as dementia, seizure, etc. Additional testing for specific frequency correlation to signs and symptoms requires a much larger homogenous population and more sensitive methods of testing than were utilized in this study. The previous study demonstrated that stimulation at 2.5 Hz and 5.5 Hz was likely associated with changes in neuronal migration and myelination [13]. Therefore, much work is needed to link a specific identified brain frequency with a function on the macroscopic or microscopic level.

Opposite polarity at each frequency, for example, 7.5 Hz versus 7.7 Hz, may represent subtle differences in misalignment in the neural circuitry at the molecular and cellular level [17]. These subtle differences have yet to be elucidated. Moreover, the authors analyzed frequency at the tenth-of-a-Hertz level, such as 7.1 Hz, but did not examine finer resolutions at the hundredth (e.g., 7.11 Hz) or thousandth (e.g., 7.111 Hz) of a Hertz. Future studies should explore EMF stimulation at these higher resolutions to clarify its modulatory effects at the organelle, cellular, and neuronal levels, which may influence cognition, behavior, and emotion. As primary brain injury is often irreversible, the management of severely brain-injured patients focuses on preventing secondary injury processes and optimizing conditions [19]. Secondary injury, particularly neuroinflammation, disrupts cellular homeostasis and promotes widespread neuronal degeneration [20,21]. Chronic brain disease is manifested by an EMF signal with fewer peaks and valleys between 4.0 Hz and 12.0 Hz being more rounded, with less amplitude, and having the first derivatives flatter within the EMF between 4.0 Hz and 12.0 Hz. However, due to the body’s ability to repair itself and increased basic cellular activity, there is more activity, more amplitude, peaks and valleys, and changes in the first derivative between 1.0 Hz and 4.0 Hz. There are certain basic functions that exist at 1-4 Hz, as evidenced by the lowest valley of the EMF in chronic brain disease patients. These identified abnormalities can be precisely stimulated to enhance that basic cellular function. In acute disease, where the injured brain function still exists as evidenced by EMF peaks and valleys corresponding to the function, the EMF appears with less amplitude, not flatter, but more of a valley. The EMF peak is still present in the OS. The idea to precisely obtain the location and frequency of the brain function in question is to diagnose the issue with specific EMF stimulation to enhance or restore that activity with positive stimulation at that frequency [8]. Identifying the specific brain, circuit, cellular, or genetic EMF activity may help to diagnose and modulate to counteract the harmful effects of inflammatory cytokines, aberrant cell signaling, and cortical spreading depressions, thereby enabling neural recovery [1,14,17,22].

The authors identify previously non-described intrinsic brain frequencies, from specific intracranial areas, corresponding to functional and anatomical changes that can be utilized for precise treatment. A modern review of pulsed EMFs (PEMF), their technology, cellular and molecular reactions, and their effects in the clinic is detailed by Flatscher et al. [23]. Modern PEMF uses coils to deliver a large magnetic field of variable waveforms, from sine waves to square waves and others of a single frequency, through a series of EMF pulses. The current study first locates brain functional and anatomical EMF signals and abnormalities, the corresponding frequencies, and the abnormal frequencies. With this information, stimulators then target the precise area with the specific frequency to induce a change and record that change. This is unlike PEMF, which is nonspecific and does not diagnose a specific pathology found in the EMF of the brain. It was critical to determine the optimal FOI for brain EMF stimulation because the goal of EMF stimulation was to achieve the most favorable therapeutic response. A favorable therapeutic response would be an improvement in symptoms such as headache, vertigo, dizziness, slow reaction time, confusion, and others, but the most favorable therapeutic response would be a complete resolution of symptoms. The corresponding favorable therapeutic electromagnetic response would be a less negative amplitude after stimulation, but the most favorable therapeutic electromagnetic response would be a positive peak (a positive amplitude) after stimulation. In two cases, there was more than one FOI found in the SOI and OS. One patient had two FOIs, and it was unclear which FOI corresponded to the patient’s clinical symptoms or neurological deficits. Therefore, 8.6 Hz was selected as the first targeted FOI. If the patient did not show improvement clinically and electromagnetically at this targeted FOI after EMF stimulation, then 11.6 Hz would be the next targeted FOI. Similarly, another patient had three FOIs found in his SOI and OS. 7.9 Hz was chosen as the targeted FOI; however, if the patient did not show improvement, then the next targeted FOI would be 9.5 Hz followed by 11.4 Hz. This process of trial and error should improve as more knowledge is gained through understanding the significance of each frequency. Currently, there is limited knowledge of the significance behind each frequency. This is an area of active research. The therapeutic effects of EMF modulation have previously been investigated in animal models simulating traumatic brain injury, spinal cord injury, and global transient stroke [12-16]. In a swine model with controlled cortical impact, it was established that EMF stimulation using thresholds of 2.5 Hz and 5.5 Hz (with a positive 500 mV offset at 1.0 V) resulted in an improvement of EMF patterns on treatment day 7 [13]. Progress has been made in Yucatan mini-swine models with induced traumatic brain injury that showed changes in gene and protein expression [17]. Future studies should investigate changes in gene and protein expression at each frequency.

While EMF can offer insight into structural and functional alterations in an injured human brain, these changes are captured as a snapshot - similar to a spot EEG, which records a single moment of brain activity. The current model captures EMF data continuously as the physiological changes occur at the time of EMF data collection. The brain’s physiology is dynamic and constantly changing to repair damages. Continuous EMF recording may reveal additional FOI, and these may represent disease in electrochemical flow, which can be used to guide EMF stimulation. After EMF stimulation, which induces changes in the EMF patterns, post-stimulation EMF data may reveal new FOI that can be targeted for treatment, and so on. In previous studies (unpublished), the authors have observed the revelation of new FOIs after EMF stimulation, and these FOIs were used to guide further EMF stimulation, resulting in favorable responses such as improvement in clinical symptoms and neurological deficits.

Limitations

While the study’s results are encouraging, limitations of this study include a small sample size, which limits generalizability, and a lack of long-term follow-up data. Further studies with larger cohorts and randomized controlled trials are required to validate these findings and to establish optimal parameters for EMF stimulation, including choice of voltage and duration of treatment.

## Conclusions

EMF of the human brain reveals frequency changes in brain, circuit, neuronal, and organelle activities in atraumatic and traumatic brain injury patients. This information can be used for targeted EMF stimulation to modulate the changes in activities to stabilize, enhance, or restore EMF activity. The frequency selection technique enables a more precise understanding of the changes that occur in brain injury, whether on a global, circuit, cellular, or organelle level. Once identified, these changes can be utilized and tailored for a more effective treatment plan. Further research is required to validate these findings.
